# Lead, Cadmium, and Arsenic in Raw Milk Produced in the Vicinity of a Mini Mineral Concentrator in the Central Andes and Health Risk

**DOI:** 10.1007/s12011-023-03838-2

**Published:** 2023-09-15

**Authors:** Doris Chirinos-Peinado, Jorge Castro-Bedriñana, Elva Ríos-Ríos, Gianfranco Castro-Chirinos, Yubaly Quispe-Poma

**Affiliations:** 1 Research Center in Food and Nutritional Security, Universidad Nacional del Centro del Perú, Huancayo, Junín Perú; 2 Department of Chemistry, Science Faculty, Universidad Nacional Agraria La Molina, Lima, Perú; 3 Psychology area, Teleperformance Peru, Lima, Perú; 4 Zootechnical Faculty, Universidad Nacional del Centro del Perú, Huancayo, Junín Perú

**Keywords:** Dietary risk quotient, Hazard quotient, Lead, Cadmium, Arsenic, Bovine milk quality

## Abstract

The bovine milk quality, safety, and security are of great concern mainly due to the dispersion of toxic substances from various anthropogenic activities and poor practices for organophosphates in agriculture use. This study evaluated the potential risk to human health from lead (Pb), cadmium (Cd), and arsenic (As) from the consumption of milk produced in an area of the Central Andes valley near a mini mineral concentrator by estimating the weekly intake (WI), dietary risk quotient (DRC), hazard quotient (THQ), and hazard index (HI) for the Peruvian population aged 2 to 85 years, in three scenarios of milk consumption by age (minimum, average, and maximum). Toxic element quantification was performed by flame atomic absorption spectrometry following standardized procedures. The mean amount ± standard deviation of Pb, Cd, and As in soils was 292±60.90, 3.54±1.58, and 5.60±2.20 mg/kg, the order of importance being Pb>As>Cd. The contents of Pb, Cd, and As in pastures were 23.17±10.02, 0.25±0.57, and 0.06±0.09 mg/kg, being from highest to lowest Pb>Cd>As. The means of Pb, Cd, and As content in 19 milk samples were 0.029±0.022, 0.007±0.006, and 0.010±0.004 mg/kg. Pb and Cd exceeded the maximum permissible limits (MPL), and the As was below the MPL. At all ages and milk consumption levels, the WI for Pb and Cd were below the estimated tolerable intake (TWI). The WI for As in < 19 years was higher than the TWI. The DRC for Pb and Cd at all three milk intake levels and all ages was < 1, and for As, it was > 1 in < 19 years, being the risk group. The TQH and HI for Pb and Cd were also > 1, signifying no health risk, and for As, the values were > 1 in < 11 years. Our results are valuable for preventing adverse health impacts from safe and innocuous milk consumption.

##  Introduction

As in any dairy production system in the world, in Peru, the first link in the dairy production chain is the dairy farmers, whose number, according to the IV Agricultural Census of 2012, amounts to 452,218 families dedicated to the production of raw milk, with small-scale production prevailing, where 86% of the production units correspond to small producers with herds of less than ten head of cattle. Of these, about 6%, which produce 50% of national production, are suppliers of industrial companies, and the difference goes to artisanal plants and direct consumption as fresh milk. Raw milk production takes place in all regions of the country, concentrated in the basins of Cajamarca, La Libertad, Lima, Ica, Junín, Arequipa, Moquegua, and Tacna [1].

In 2021, per capita dairy consumption was 83.5 kg [2], being a country of intermediate consumption, of which 28.7 kg corresponds to fresh milk; this consumption will increase in the following years, which will require better production practices and quality control of raw milk to sustain the growth of dairy production and the demand that the growing population will demand.

Anthropogenic pollution from numerous farms has caused changes in the natural composition of soils and vegetation [3], entering the food chain and affecting human health [4, 5]. In humans, heavy metals are the most dangerous toxicants with direct and negative synergistic effects [6], whose density is > 5 g/cm^3^ or their relative atomic mass is > 40. Within this group, the most dangerous are cadmium (Cd) and lead (Pb) [7], and arsenic (As) is an inevitable contaminant metalloid for humans due to its multiple forms of exposure [8]. These elements emitted by various industries pollute the air and reach water and soils, plus those deriving from the use of fungicides, phosphorus fertilizers, and wastewater or contaminated water pass into the food chain [5, 9, 10]. Pb that accumulates in soil and plants originates mainly from fine particulate matter emitted by the mining-metallurgical industry, exhausts gases from internal combustion engines, and has a toxic effect on plants, animals, and humans, even at low concentrations [11]. Cd is another metal of high toxicity to soil microorganisms, plants, animals, and humans, and generally comes from the widespread use of phosphate fertilizers. A maximum concentration of Cd is in the range of 0.2–2.0 mg/kg soil, depending on the current soil fertility level [12].

Arsenic is another highly toxic element known since ancient times and is considered a carcinogen [13, 14]. It affects systems and organs, including the skin, respiratory, cardiovascular, immune, genitourinary, reproductive, digestive, nervous, erythropoietic, endocrine, hepatic, and renal systems [15].

The Mantaro Valley, located in the Central Andes of Peru, is exposed to contamination not only from emissions from the metallurgical complex of La Oroya, whose vapors and dusts travel in the air for many kilometers, but also from traffic, cement industries, and misuse of phosphorus products in agriculture and contaminate water and soil [16], a context observed in many developing countries. Therefore, products obtained from contaminated soils should be analyzed for the contents of heavy metals and other highly toxic substances and the risk of their consumption for humans. The study objective was to determine the concentration of Pb, Cd, and As in whole milk from cows raised in an area adjacent to a mineral concentrator in Huancayo province in central Peru and to evaluate the risk for the population aged 2 to 85 years. For this purpose, the weekly intake (WI), the risk coefficient (TQH), and the hazard index (HI) were estimated to have evidence to demonstrate from a toxicological point of view if this milk is suitable for human consumption based on Codex Alimentarius and other international standards and to prevent the adverse effects of these heavy metals and the health risk [17–20].

## Materials and Methods

### Ethical Approval

The work was approved by the Instituto Especializado de Investigación de la Facultad de Zootecnia of the Universidad Nacional del Centro del Perú, and the research conforms to the ethical principles of research.

### Location and Study Period

The milk sampling and quantification of Pb, Cd, and As were carried out in March 2022 in a semi-industrial dairy cattle raising area in the district of El Tambo, province of Huancayo, Peru, at 3214 m.s.n.m., south latitude 12° 4′ 36.3″ S (−12.07675254000) and west longitude 75° 13′ 30.2″ O (−75.22505777000). The farm is located on the urban periphery on the right bank of the Mantaro River, and its pastures adjoin a mini mineral concentrator that has been operating for more than five decades. The climate of the study area is characterized by two well-defined seasons. The rainy season is from October to March, and the dry season is from April to September. According to Köppen and Geiger, the climate is classified as ET. The average annual temperature and rainfall are 8.7°C and 1682 mm.

### Animals and Breeding System

Brown Swiss cows were between 3 and 8 years old, with a mean of 4.5 years, with an average production of 9 l/day; raised in a semi-extensive system with daily grazing with access to watering troughs and *Dactylis glomerata* paddocks, plus a ration of alfalfa (*Medicago sativa*) and barley (*Hordeum vulgare*) forage at cutting, all produced on the same farm. The pastures were irrigated with water from a spring near the Mantaro River and used as animal drinking water.

The cows belong to the most representative farm in the area, from which the rest of the breeders adopt their technology since they perform technological transfer to the small neighboring farms, ensuring the representativeness of the samples from the area of the study site.

### Milk, Soil, and Pasture Sampling and Heavy Metal Analysis

Once milking was completed, 19 samples of 250 mL of milk were collected. The milk samples were obtained according to the protocol of the Peruvian Technical Standard 202.112:1998 revised in 2013, using sterile polyethylene bottles of first use previously washed with nitric acid and rinsed with double-distilled water, being labeled and kept in a cold chain (4°C) for immediate shipment to the Pacific Control laboratory, Lima, Peru, certified by the National Institute of Quality - INACAL - PERU.

The samples were prepared by dry and acid mineralization; 50 g of each homogenized sample was placed in porcelain crucibles to be dried at 100°C until reaching a constant weight and was incinerated in a muffle furnace (Protherm 442-ECO110/15) at 450° C/15 h, and after cooling to 15–18°C, the contents were bleached with 2 mL of HNO_3_ 2 N, evaporating the acid on thermostatic plates and after cooling were again incinerated at 450 C/1 h. The ashes were recovered with 20 mL of 0.1 N HNO_3_, filtered through Watman 40 paper, and stored in polypropylene tubes under refrigeration. High-purity reagents (Merck KGaA, Darmstadt, Germany) were used.

Then, the spectrometer was prepared, and the measurement conditions were selected [21]. For Pb and Cd quantification, the AOAC 973.35 method was followed, using a flame atomic absorption spectrometer (NAMBEI AA320N), with wavelengths of 283.3 and 228.8 nm for Pb and Cd [22]; for As, the wavelength was 193.7 nm [23]. To determine the total As concentration in the milk, the samples were evaporated to dryness with concentrated hydrochloric acid addition. The dried residues were incinerated in a muffle furnace at 450°C [24].

To provide complementary information of Pb, Cd, and As concentration was determined in 6 soil and grass samples from the same sampling site. 0.5-kg samples of the topsoil (0–20-cm depth) were collected using standardized procedures [25]; after 1 day of natural drying, they were crushed and sieved (2-mm mesh) eliminating gravel, stones, and other impurities; they were homogenized in a mortar, weighed, and placed in airtight bags to be sent to the laboratory, where the USEPA 3050B (SW-846) method was followed, by digesting 1 g of dry sample treated with HNO_3_ (Sigma-Aldrich, USA) and H_2_O_2_. HCl acid (Sigma-Aldrich, USA) was added to the initial digest and was heated to reflux to increase the solubility of the metal. The digestion product was diluted to a final volume of 100 mL [26], and the metals were quantified by flame atomic absorption spectrometry (NAMBEI AA320N), following the AOAC Official Method 975.03 protocol [26, 27]. The corresponding grass samples were washed with tap water, removing soil particles, rinsed with deionized water [28], dried at 70°C, and finely ground [29]. The digestion and quantification procedure of the heavy metals of the pastures was similar to that of the soil.

The analysis conditions of each element used in this study respond to protocols used by a laboratory accredited by the National Institute of Quality of Peru, which uses validated analytical methods. Duplicate samples allowed for determining the precision method and calculating the mean and the coefficient of variation, which was less than 5%. Precision is measured using standard solutions of each element, determining the relative error, which in percentage represents the precision method and must exceed 95%. For these calculated used standard solutions of Pb, Cd, and As of 155, 150, and 50 mg/kg of milk [23], at analysis, the corresponding concentrations were 148.14, 152.50, and 49.58 mg/kg, values that transformed to percentage indicate that the method complies with the precision parameters.

To the calibration curves, standards (Merck) of 1000 mg/kg were used for each element. Limits of detection (LOD) to Pb, Cd, and As in milk were 0.03, 0.03, and 0.028 μg/L, respectively. The LODs for the forage were 2.40, 0.40, and 0.03 μg/kg, and the LODs for the soil were 0.1, 0.01, and 0.02 mg/kg, respectively. The concentrations of heavy metals in all the study samples are expressed in mg/kg, and values that for the calculations of weekly intake, objective risk, and risk index were transformed to μg/kg. To evaluate the concentration of Pb and Cd in the soil, the Standard Environmental Quality Standard of 70 and 1.4 mg/kg, respectively [30], was considered. For As, the Canadian Council of Ministers of the Environment [31, 32] indicates that the maximum concentration in the soil is 12 mg/kg. The maximum suggested Pb limit for forage is 10 mg/kg of dry matter [33–35]; for cadmium and arsenic in livestock feed, the Council of the European Parliament indicates values of 1 and 2 mg/kg [36].

### Estimated Weekly Intake (WI)

The heavy metal exposure of the population (from infants to the elderlies) was estimated using the average concentrations of Pb, Cd, and As in milk from the study area. To obtain a complete figure of the relative risk in the sampled area, we conducted a literature survey to identify the average body weight of the Peruvian population at different ages [37], using a study that evaluated 62600 people (<10 *n*=12327, 10–19 *n*=14597, 20–29 *n*=7632, 30–39 *n*=7832, 40–49 *n*=7381, 50–59 *n*=5613, 60–69 *n*=3816, >70 *n*=3402), being to date the only study reporting results for the Peruvian population aged 2–85 years. Exposure to metals from milk consumption was considered chronic. Weekly intakes of Pb, Cd, and As from consumption of raw cow’s milk in three scenarios: low, medium, and high consumption were determined [9, 38, 39].

The weekly intake of each metal (WI: μg/week of milk consumption) was estimated by comparing it with the tolerable weekly intake (TWI) established for Pb and Cd [19, 20, 40, 41] and As [42].

In this study, the daily milk intakes considered as minimum, average, and maximum levels in children aged 2–5 years are 400, 500, and 600 g/day. Children aged 6–19 were 500, 600, and 720 g/day. For people aged 20–85 years, the intakes were significantly lower, between 100 and 250 g/day [43–48]. For this purpose, a table of milk intake was prepared for ages 2 to 85 years, with continuous data for the corresponding risk calculations.

### Dietary Risk Coefficient (DRC)

The following formula was used to estimate the dietary risk coefficients (DRC):

$$\textrm{DRC}=\textrm{WI}/\textrm{TWI}$$

where DRC is the dietary risk coefficient. WI is the amount of metal ingested during 1 week from milk consumption (μg/week). TWI is the tolerable weekly intake of the metal (μg/week). A TWI below 1 indicates an acceptably low risk, while a ratio above 1 indicates a high health risk [49, 50].

### Chronic Potential Risk (TQH) and Hazard Index (HI)

The TQH and HI for Pb, Cd, and As, for average milk consumption, demonstrate from a toxicological point of view whether the milk produced in the study area is within the levels established by Codex Alimentarius [17] and another international standard and avoids the adverse effects of these heavy metals and the risk to human health ([18–20].).

To assess the TQH for human health for metals associated with long-term exposure from the consumption of raw cow milk with Pb, Cd, and As, the following equation was used [51]:

$$\textrm{TQH}=\frac{\left(\textrm{EF}\ast \textrm{ED}\ast W\textrm{milk}\ast C\textrm{metal}\right)}{\textrm{RfD}\ast \textrm{Body}\ \textrm{Weight}\ast \textrm{TA}}$$

where *C*_metal_ is the metal content in milk. *W*_milk_ is the daily milk consumption. EF is the exposure frequency (365 days per year). ED is the period of exposure equivalent to the average longevity for an adult. For Peru, it is estimated at 76.5 years. AT is the average useful lifetime, which is 27922.5 days. RfD is the reference oral dose. For Cd, Pb, and As, values of 0.001, 0.0035, and 0.0003 mg/kg b.w./day were used, respectively [52–54].

The HI was used to assess the potential chronic risk to human health when involving several heavy metals and represents the long-term risk; it was determined by the sum of the THQs for Pb, Cd, and As [4, 9, 55]. If HI is < 1, there is no risk to human health [56]. Target hazard quotient (THQ) and hazard index (HI) values for As, Pb, and Cd from milk consumption were calculated for individuals aged 2 to 85.

### Data Processing Technique

The concentrations of the elements detected in the samples were calculated in mg/kg. To evaluate if the average contents of Pb, Cd, and As in the soil, forage, and milk exceed the permitted limits, one-sample “*t*” tests were used. The maximum permissible limits (MPL) of Pb used for the soil, forage, and milk were 70, 30, and 0.02 mg/kg, respectively; for Cd, the values were 1.4, 1.0, and 0.0025, and for As, the values were 12.0, 2.0, and 0.014 mg/kg, respectively. For statistical comparisons between the concentrations of each element in the soil, forage, and milk samples, one-way ANOVAs were performed. Pearson correlations were determined for elements’ associations between soil-forage and forage-milk. Differences between means were assessed using Tukey’s test, and *P* values < 0.05 were considered significant. Weekly intake calculations and health risk coefficients were estimated in μg/kg and processed SPSS V26 (IBM, Endicott, NY, USA).

## Results

### Pb, Cd, and As Content in the Study Samples

The mean amount of Pb, Cd, and As in the soils of alfalfa, dactylis, and barley crops used in cow feed in importance order was Pb>As>Cd. The contents of heavy metals in the pastures, from highest to lowest, were Pb>Cd>As (Table 1), and raw milk contents of Pb, Cd, and As are shown in Table 2.

**Table 1:** Pb, Cd, and As concentrations in soil, sprouts, and milk, and bioaccumulation percentage, in the Central Andes-2022 (mg/kg)

	Pb			Cd			As		
	Soil	Sprouts	Milk	Soil	Sprouts	Milk	Soil	Sprouts	Milk
Mean	292.00a	23.17b	0.029c	3.54a	0.250b	0.007c	5.60a	0.063b	0.010c
SD	60.90	10.02	0.022	1.58	0.57	0.006	2.20	0.09	0.004
*T*, %	100.00	7.93	0.13	100.00	7.06	2.80	100.00	1.13	15.87
*B*, %	100.00		0.01	100.00		0.20	100.00		0.18

^a,b,c^Mean values per element with different letters vary statistically (*P*<0.01)

*SD*, standard deviation

*T, %*, percentage of transfer from soil to pasture and from pasture to milk

*B, %*, percentage of bioaccumulation (soil to milk)

**Table 2:** Pb, Cd, and As concentrations in raw milk produced in the Central Andes-2022 (mg/kg)

No. of cow	Lead	Cadmium	Arsenic	Total
1	0.054	0.023	0.022	0.099
2	0.014	0.004	0.010	0.028
3	0.058	0.017	0.010	0.085
4	0.014	0.003	0.009	0.026
5	0.075	0.008	0.015	0.098
6	0.015	0.005	0.010	0.030
7	0.056	0.015	0.008	0.079
8	0.014	0.004	0.008	0.026
9	0.068	0.009	0.017	0.094
10	0.015	0.003	0.008	0.026
11	0.013	0.002	0.010	0.025
12	0.012	0.004	0.008	0.024
13	0.030	0.005	0.007	0.042
14	0.040	0.004	0.010	0.054
15	0.013	0.003	0.007	0.023
16	0.020	0.005	0.009	0.034
17	0.020	0.004	0.009	0.033
18	0.014	0.004	0.011	0.029
19	0.013	0.003	0.009	0.025
Mean	0.029a	0.007b	0.010b	0.046
SD	0.022	0.006	0.004	0.032
MPL	0.020	0.0025	0.014	

^a,b^Mean values with different letters vary statistically (*P*<0.01)

*MPL*, maximum permissible limit

The linear correlation coefficient is a regression measure used to establish a linear relationship between two variables and quantify the degree of joint variation between them. A strong positive linear relationship was observed between the concentrations of Pb and Cd (*P*=0.001, *r*=0.716), Pb and As (*P*=0.004, *r*=0.625), and Cd and As (*P*=0.003, *r*=0.642). As the concentration of Pb increased, the concentration of Cd and As also increased.

Although it is not the objective of this study to analyze the dynamics of these elements in the soil-grass-milk system, it can be indicated that the percentage of bioaccumulation in order of importance is Cd>As>Pb.

### Estimated Daily Intake (WI) and Dietary Risk Coefficient (DRC)

**Fig. 1:**
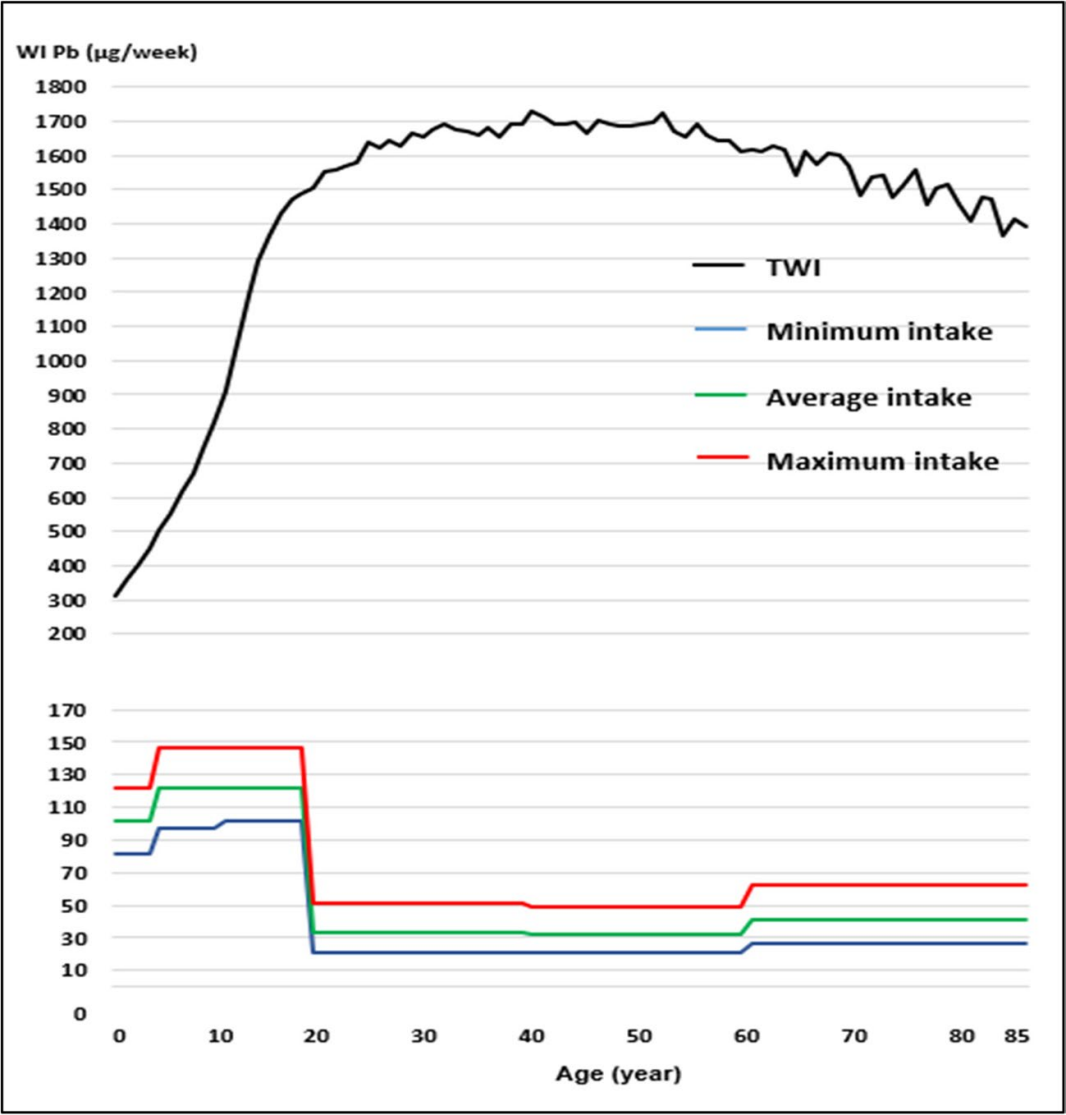
Weekly intake curve (WI) of Pb from milk consumption in people aged 2–85 years, at minimum, average, and maximum exposure

**Fig. 2:**
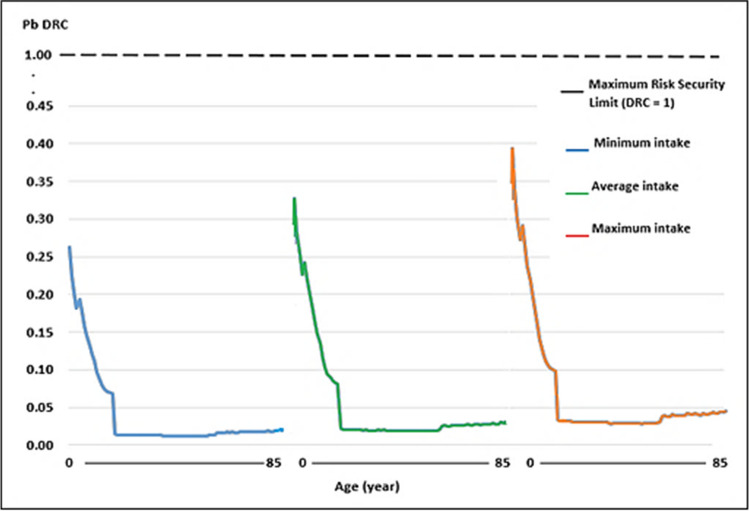
Dietary risk coefficient (DRC) to Pb from milk consumption in people aged 2–85 years, at minimum, average, and maximum exposure

**Fig. 3:**
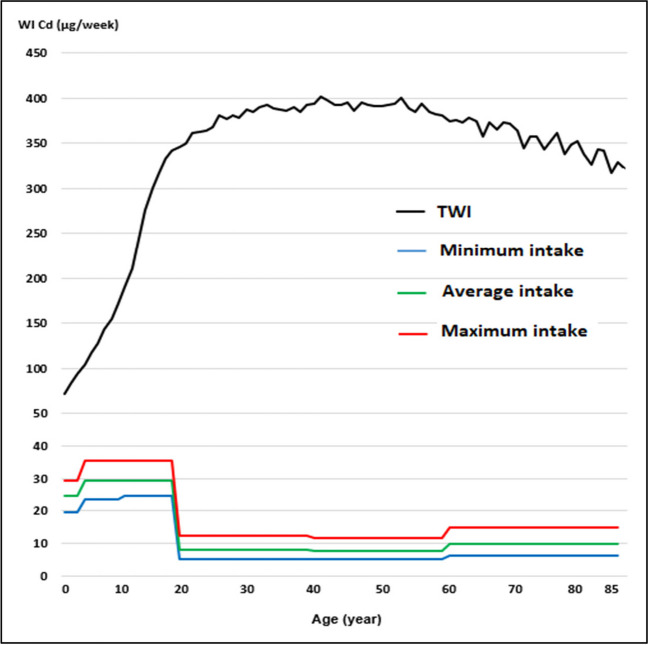
Weekly intake curve (WI) of Cd by milk consumption in people aged 2–85 years, at minimum, average, and maximum exposure

**Fig. 4:**
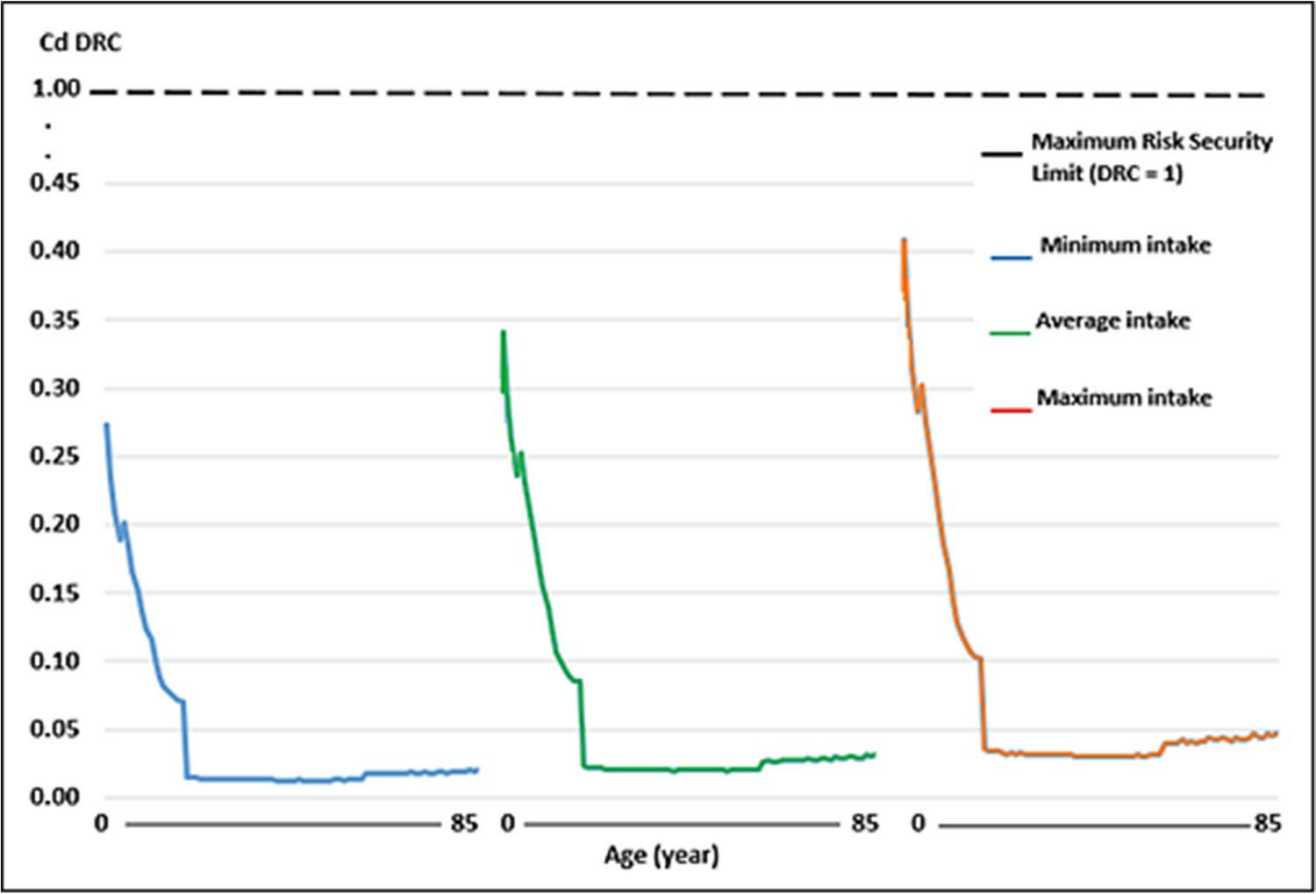
Dietary risk coefficient (DRC) to Cd from milk consumption in people aged 2–85 years, at minimum, average, and maximum exposure

**Fig. 5:**
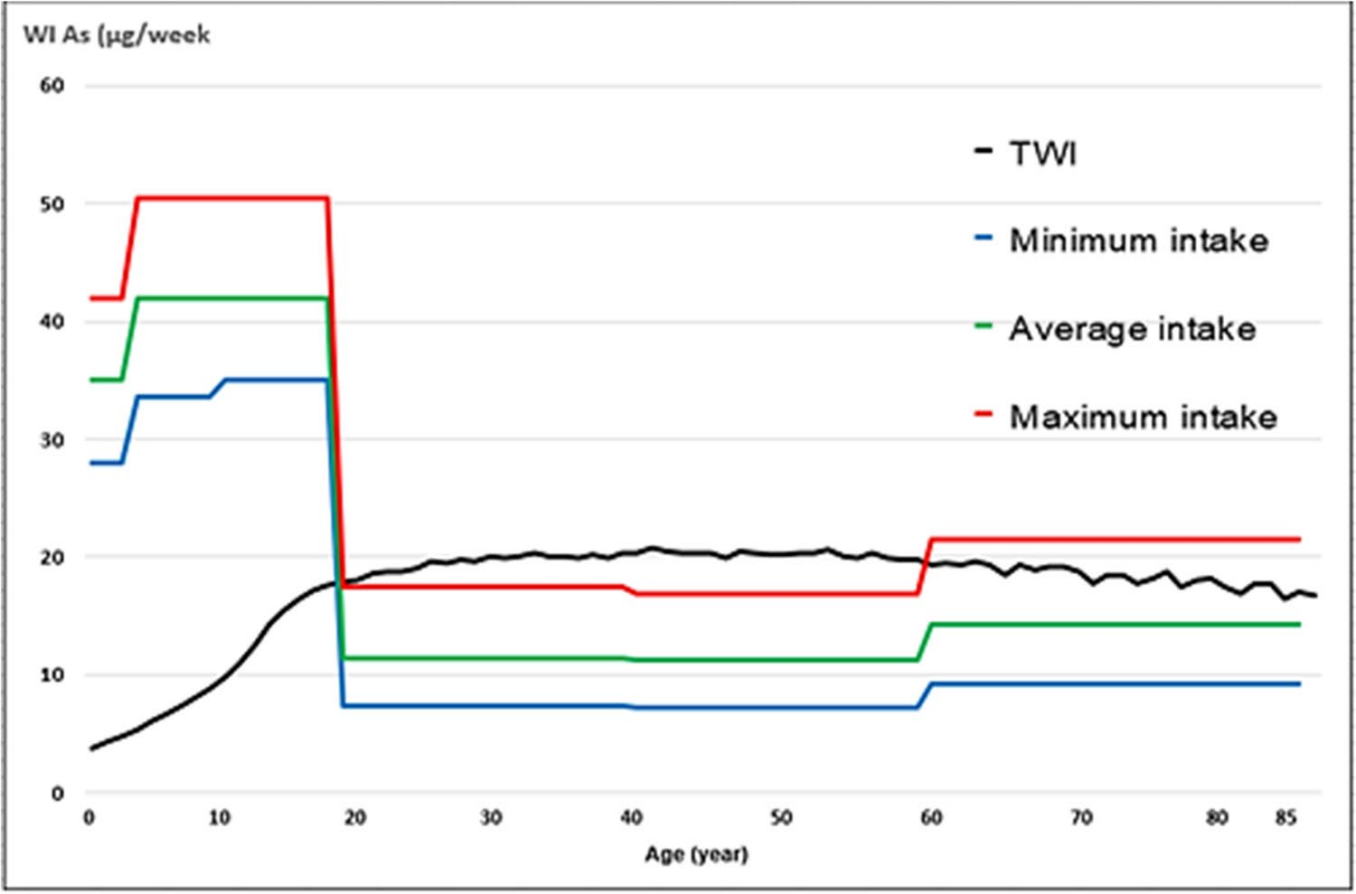
Weekly intake curve (SI) of As from milk consumption in people aged 2–85 years, at minimum, average, and maximum exposure

**Fig. 6:**
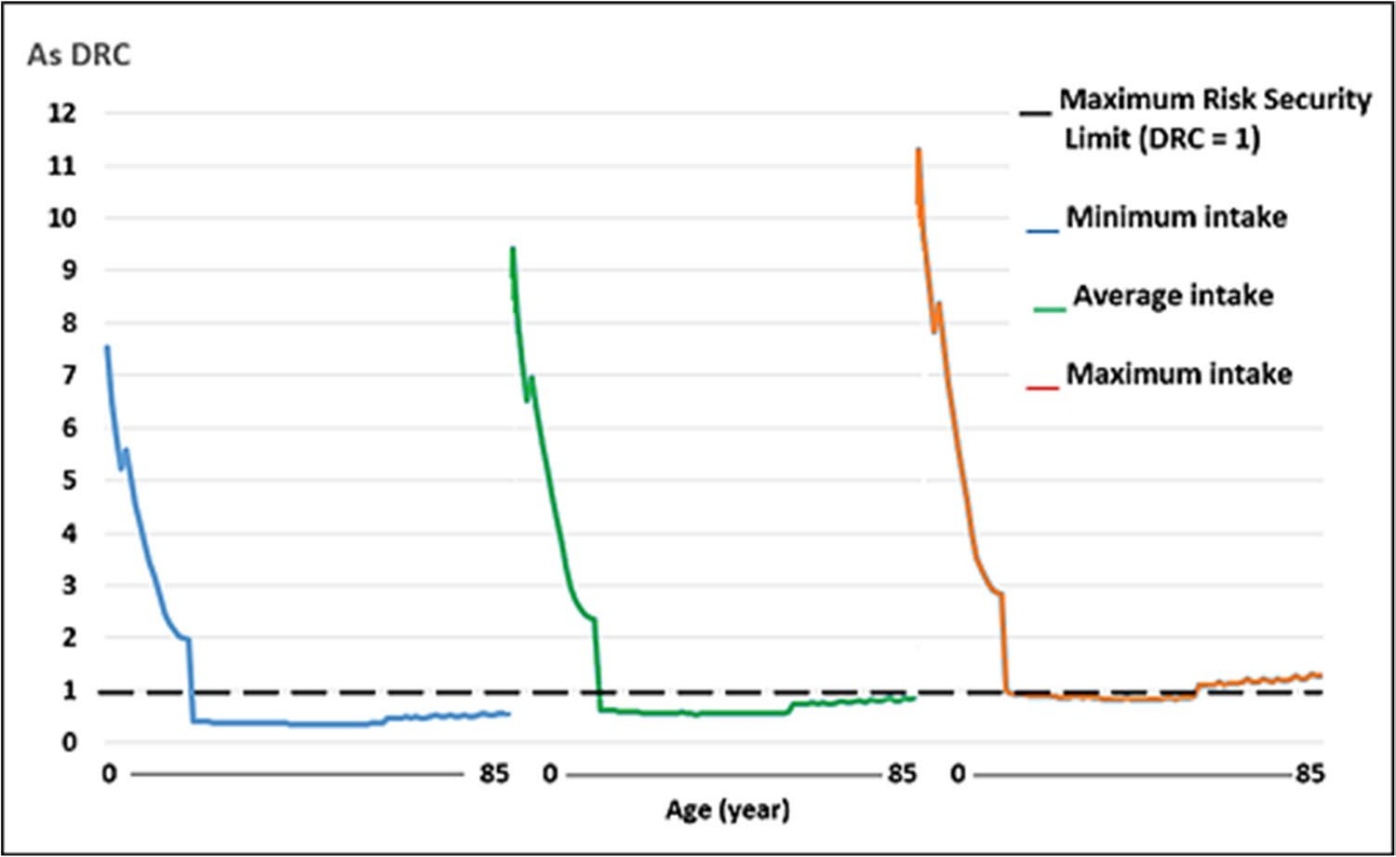
Dietary risk coefficient (DRC) to As from milk consumption in people aged 2–85 years, at minimum, medium, and maximum exposure

Regarding the WI of Pb and Cd, by consumption of raw cow’s milk, the values were well below the TWI established by the Joint FAO/WHO Expert Committee on Food Additives [41] (Figs. 1, 2, 3 and 4). Whereas for As, the WI for people aged 2 to 19 years was well above the TWI established by USEPA [42], and in people older than 60 years, with the maximum milk intake, it was also slightly above the TWI (Figs. 5 and 6).

### Target Hazard Coefficient (TQH) and Risk Index (HI)

The THQ followed a descending order of As > Pb > Cd, with values of 0.05–1.13, 0.01–0.28, and 0.01–0.24 for minimum milk intake in persons aged 2–85 years, 0.09–1.41, 0.02–0.35, and 0.02–0.30 for average intake, and 0.13–1.69, 0.03–0.42, and 0.03–0.36 for maximum milk intake, with higher values at lower ages. Table 3 shows, as an example, the parameters used in the calculations for the minimum consumption scenario of milk. Similar data for average and maximum intake were generated.

**Table 3:** Parameters used in target hazard quotient (THQ) and hazard index (HI) determination in the minimum consumption scenario of milk contaminated with Pb, Cd, and As

Age (year)	Weight (kg)	Milk intake (kg/day)	Pb exposure/day	Cd exposure/day	AS exposure/day	WI Pb	WI Cd	WI As	TWI Pb	TWI Cd	TWI As	DCR Pb	DCR Cd	DCR As	TQH Pb	TQH Cd	TQH As	HI
2	12.40	0.400	11.6	2.80	4.00	81.20	19.60	28.00	310	72	4	0.26	0.27	7.53	0.28	0.24	**1.13**	**1.65**
3	14.40	0.400	11.6	2.80	4.00	81.20	19.60	28.00	360	84	4	0.23	0.23	6.48	0.24	0.20	0.97	**1.42**
4	16.10	0.400	11.6	2.80	4.00	81.20	19.60	28.00	403	93	5	0.20	0.21	5.80	0.21	0.18	0.86	**1.25**
5	17.90	0.400	11.6	2.80	4.00	81.20	19.60	28.00	448	104	5	0.18	0.19	5.21	0.19	0.16	0.77	**1.12**
6	20.10	0.480	13.9	3.36	4.80	97.44	23.52	33.60	503	117	6	0.19	0.20	5.57	0.20	0.17	0.82	**1.20**
7	22.00	0.480	13.9	3.36	4.80	97.44	23.52	33.60	550	128	7	0.18	0.18	5.09	0.19	0.16	0.75	**1.09**
8	24.60	0.480	13.9	3.36	4.80	97.44	23.52	33.60	615	143	7	0.16	0.16	4.55	0.17	0.14	0.67	0.97
9	26.80	0.480	13.9	3.36	4.80	97.44	23.52	33.60	670	155	8	0.15	0.15	4.18	0.15	0.13	0.60	0.87
10	29.60	0.480	13.9	3.36	4.80	97.44	23.52	33.60	740	172	9	0.13	0.14	3.78	0.13	0.11	0.54	0.78
15	51.70	0.500	14.5	3.50	5.00	101.5	24.50	35.00	1293	300	16	0.08	0.08	2.26	0.08	0.07	0.34	0.49
20	60.30	0.096	2.8	0.67	0.96	19.49	4.70	6.72	1508	350	18	0.01	0.01	0.37	0.02	0.01	0.07	0.10
25	65.60	0.096	2.8	0.67	0.96	19.49	4.70	6.72	1640	380	20	0.01	0.01	0.34	0.02	0.01	0.06	0.09
30	66.30	0.096	2.8	0.67	0.96	19.49	4.70	6.72	1658	385	20	0.01	0.01	0.34	0.01	0.01	0.06	0.09
40	69.30	0.094	2.7	0.66	0.94	19.08	4.61	6.58	1733	402	21	0.01	0.01	0.32	0.01	0.01	0.06	0.08
55	67.80	0.094	2.7	0.66	0.94	19.08	4.61	6.58	1695	393	20	0.01	0.01	0.32	0.01	0.01	0.06	0.08
60	64.80	0.120	3.5	0.84	1.20	24.36	5.88	8.40	1620	376	19	0.02	0.02	0.43	0.02	0.02	0.07	0.11
65	64.40	0.120	3.5	0.84	1.20	24.36	5.88	8.40	1610	374	19	0.02	0.02	0.43	0.02	0.02	0.08	0.11
70	59.30	0.120	3.5	0.84	1.20	24.36	5.88	8.40	1483	344	18	0.02	0.02	0.47	0.02	0.02	0.08	0.12
75	62.30	0.120	3.5	0.84	1.20	24.36	5.88	8.40	1558	361	19	0.02	0.02	0.45	0.02	0.02	0.08	0.12
80	56.30	0.120	3.5	0.84	1.20	24.36	5.88	8.40	1408	327	17	0.02	0.02	0.50	0.02	0.02	0.08	0.12
85	55.60	0.120	3.5	0.84	1.20	24.36	5.88	8.40	1390	322	17	0.02	0.02	0.50	0.02	0.02	0.09	0.13

This table shows the parameters used to determine the WI, DCR, TQH, and HI for continuous data from the Peruvian population aged 2–85 years in the minimum consumption scenario of milk. For having a summary table, from 10 years onwards, data is shown every 5 years. TQH and HI values in bold type exceed the value of 1, indicative of risk to human health at those ages

**Fig. 7:**
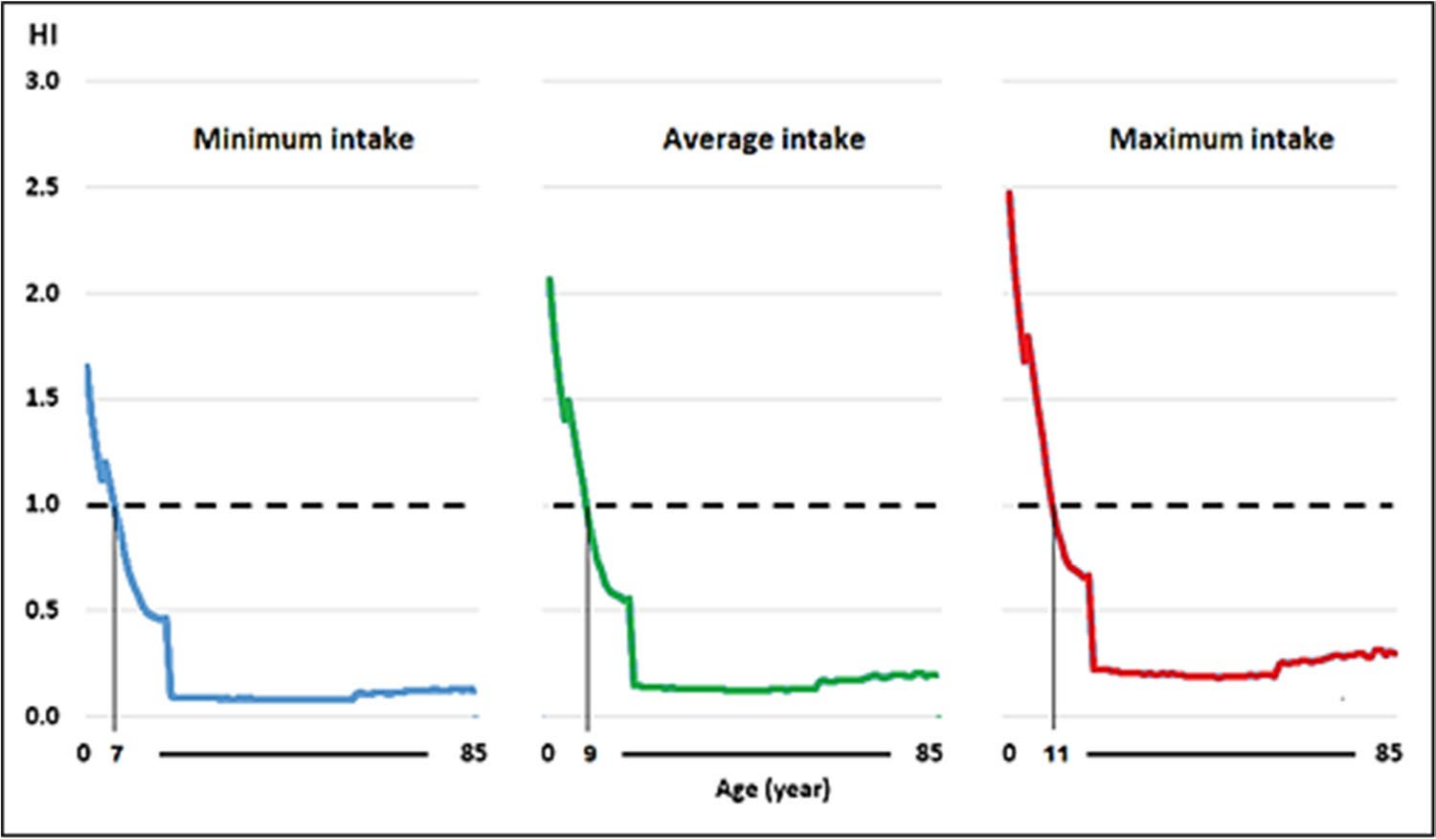
Risk index (HI) for minimum, average, and maximum milk consumption contaminated with Pb, Cd, and As in the Peruvian population aged 2 to 85 years

The HI values for low, average, and maximum milk consumption ranged between 0.08–1.65, 0.13–2.06, and 0.19–2.47, respectively, with values > 1 being observed in children under 7, 9, and 11 years of age for milk consumption at the minimum, average, and maximum levels. In older people, all values were well below 1 (Fig. 7). Pb, Cd, and As contents of 29, 7, and 10 μg/kg were considered for calculations. This information is useful and allows us to differentiate exposure to these elements by age, weight, and the risk of consuming milk produced in the mini mineral concentrators’ vicinity.

## Discussion

The increase in anthropogenic activities (industry, mining, agriculture) also increases the emission of pollutants into ecosystems. Dairy farms near these industries are affected by heavy metals presence that accumulate in the soil, pastures, and animals and are eliminated through organs and tissues, especially in milk which is consumed by humans. In this study, it is evident that the heavy metal’s main source of exposure is the farm’s proximity to a mini mineral concentrator plant.

### Pb, Cd, and As Content in the Study Samples

The concentration of Pb in the upper layer of the soil was four times higher than the MPL established for agricultural soils in Peru [30], which comes mainly from emissions from the mini mineral concentrator adjacent to the farm whose soil has bioaccumulated heavy metals for more than five decades, and transfer them to pastures whose content represented 77% of the maximum limit. These Pb contents in the soil and forages were similar to those reported in an area close to the La Oroya metallurgical complex, with 218±9.8 mg/kg and in pastures an average of 20±0.9 mg/kg [57].

Worldwide, various levels of heavy metals in the soil are reported, the values of which depend on environmental conditions, the world average being around 25 mg/kg, and it would seem that Peru is at the upper end of the world scale. Martin et al. [25], in southern New Zealand soils, report high levels of Pb and Cd in exotic grassland lands, noting that the principal source of heavy metals is the application of phosphorus-based fertilizers and proximity to urban centers and near roads. In the present study, in addition to the use of phosphorous fertilizers, the principal source would be given by the emissions from the mini mineral concentrator adjacent to the farm whose tailings leach and are deposited and accumulated in the upper layer of the soil and are absorbed by the pastures, added to those from irrigation wastewater, results that would be in line with what has been indicated by different authors [58, 59].

The Cd content in the study soils was 2.5 times higher than the MPL, in the forages represented 25% of the MPL, the potential sources of Cd being the use of synthetic fertilizers, the presence of mining-metallurgical liquid waste dumped into the Mantaro River and mainly the proximity to the mini mineral concentrator whose discharges adjoin the grazing area and its dust contaminates the soil. In various regions of the world, where there is better control of industrial emissions, lower or similar Cd contents are reported; thus, Tepanosyan et al. [12], Zhou et al. [5], and Kozhanova et al. [60] report Cd concentrations in the soil between 0.2 and 2.0 mg/kg. Martin et al. [25] report that in New Zealand, they have suggested a national average baseline of 0.16 mg/kg and 0.35 mg/kg on all agricultural land.

In this study, the As content in the soil represented 47% of the ML, and in the case of pastures, it represented 2.7% of MPL. The As content in soil was well above those reported in other latitudes; thus, Kozhanova et al. [60] in different seasons reported the As concentrations of 0.022 and 0.019 mg/kg with a range of 0.20–0.27 mg/kg, the lowest in spring and highest in autumn. Martin et al. [25] in New Zealand soils report average contents of 3.5 mg/kg, with a range of 0.4 and 10.9 mg/kg, related to the structure and type of soil that sequesters As, with variations in As levels in soils and pastures.

In Dong Mai, Hung Yen-Vietnam, Ha et al. [14] in soils of native and cultivated plants in a Pb recycling area reported lower contents of Pb and Cd (5.4–26.8 and 0.71–1.67 mg/kg) and higher As (370–47400 mg/kg) than in this study. The As, Cd, and Pb contents in rice grains and shoots of 15 plant species ranged from 0.14–10.2, 0.06–0.99, and 2.83–1160 mg/kg dry weight, respectively, while in this study, the grasses had much higher Pb contents, similar Cd contents, and lower As contents.

In the context of Peru, in Tiquillaca, Puno, a department located in southern Peru, the averages of Pb, Cd, and As in the soil were 276.74, 1.45, and 5.35 mg/kg, respectively, values similar to our study and attributed to the dumping of mining waste whose dispersion causes soil contamination [32], a problem similar to that of the present study, where the stable is adjacent to a mini mineral concentrator. In another study, Soto-Benavente et al. [61], in the Peruvian Amazon, in soils abandoned by gold mining, report values of 12.6, 0.79, and 6.67 mg/kg for Pb, Cd, and As, respectively, being much lower in Pb and Cd than our study, but higher in As and would be due to the chemical compounds used in informal gold mining.

It is important to consider that heavy metals can be deposited in agricultural soils through manure [60], pesticides, phosphorus fertilizers, and fertilizers [62], which affect the productive quality of the soil. In Peru, there are big deposits of phosphoric rock that for decades have been used directly as fertilizer or raw material for the manufacture of commercial phosphorus products that also contain residues of Cd, Cr, Zn, As, and other heavy metals. Cadmium appears in concentrations of 1–200 mg Cd/kg P_2_O_5_, and it is present in phosphate fertilizers for commercial use, and a moderate correlation (*r* = 0.66) between the application of phosphorus fertilizers and the accumulation of Cd [25].

The Pb contents in the forages used to feed the cows in the study area were higher than those reported in another cattle-raising area of the Mantaro Valley, which was 5.8 mg/kg [63], while the Cd level was ten times lower than those found in this study, and would be because the high amounts of phosphorous fertilizers and irrigation with water from a highly polluted canal.

In other regions of the world, average Cd and As contents are reported in cattle feeds, well above those found in this study; in China, 2.31 and 1.38 mg/kg of Cd and As are reported [64]; in this study, Cd represented 10% and As 4% of what was found in China; however, even when the contents of Cd and As in the pastures of this study do not exceed the MPL, the chronic intake of these elements bioaccumulates in the animal organism and passes into the milk.

In this study, the mean contents of Pb and Cd in milk exceeded the maximum limits by 1.45 and 2.8 times those allowed set at 0.02 and 0.0026 mg/kg, respectively [17, 65, 66], and even when As represents approximately 70% of the maximum allowed limit set at 0.014 mg/kg [67], this milk would not be suitable for human consumption or environmental safety. The concentration of these toxic elements in order of importance is Pb>As>Cd, with Pb, Cd, and As representing 63, 15, and 22% of the total.

Regarding the content of heavy metals in milk, Boudebbouz et al. [68], making a worldwide review of original articles published between 2010 and 2020, report Pb levels in raw cow’s milk below the detection level up to 60 mg/L in a granite and granite gneiss mining area in India. The accumulation of heavy and toxic metals in milk and milk products depends on the farm location [69], and the content of heavy metals in milk differs according to the country and the local context; thus, the MPL of Pb in cow’s milk are 0.05, 0.02, and 0.10 mg/kg, in Germany and the Netherlands, Turkey, and Russia, respectively [70]. In Iran, Shahbazi et al. [69] report 0.014 mg/kg, which represents 48% of the Pb content determined in the milk of this study.

The concentration of Cd in the study milk was lower than the values reported in Kazakhstan by Kozhanova et al. [60], from 0.01 to 0.02 mg/L, in summer and autumn, respectively, but it was higher than that reported in Iran by Shahbazi et al. [69]; [71] which was 0.0011 mg/kg and would be because the farm in our study adjoins a mini mineral concentrator plant.

The As concentration in milk also varies depending on the sampling location [13].

Ayala and Romero [72], the Technical Standard of Ecuador - NTE 0009:2008, considers the MPL 0.015 mg/kg, while the national standard of the People’s Republic of China establishes 0.10 mg/kg and the official Mexican standard NOM-184-SSA1-2002 indicates an MPL of less than 0.20 mg/kg. MERCOSUR - RES. N° 12/11 suggests 0.05 mg/kg.

Pérez-Carrera and Fernández-Cirelli [73], in milk sampled in Córdoba-Argentina, report an As content in the 0.0003–0.0105 mg/kg range. For their part, Licata et al. [74], in Calabrian farms, report a mean As content of 0.038 mg/kg, and in Arak-Iran, raw cow’s milk has As amounts below the MPL [75].

Su et al. [76], in areas close to leather processing plants in China, reported that the As and Pb milk concentrations from contaminated zones were 0.43±0.21 and 2.86±0.96 μg/L, respectively, values significantly higher than those from uncontaminated farms with 0.20±0.05 and 2.32±0.78 μg/L. The Cd level in contaminated milk zones was 0.15 ± 0.04 μg/L similar to that of uncontaminated zones with 0.13 ± 0.04 μg/L (*P* > 0.05).

As observed in different investigations, among the principal sources of animal heavy metal exposure are pastures and forages produced on the same farm, with water and soils contaminated by mining-metallurgical emissions, the uncontrolled use of chemical phosphate fertilizers, irrigation water from polluted rivers with industrial waste, among others [63].

Table 1 also shows that the percentage of soil-sprout-milk transfer is very different in the case of As versus Cd and Pb. The transfer from the soil to the pastures was similar for Pb and Cd, being approximately seven times more than for As, which would indicate that the study area pastures would have a greater capacity to accumulate Pb and Cd from the environment and through the roots translocate to the stems and leaves. Transfer levels are influenced by a variety of factors, such as the exposure level, phytoextraction capacity of plants, the physical-chemical characteristics of the soil, the type of metal, and phytoavailability, which depends on its chemical forms, among others [77, 78].

One aspect that draws attention is that the transfer of As from grass to milk was 5.7 times more than that of Cd and 122 times more than that of Pb. Due to the high levels of As found in milk, its consumption by humans would represent an important route of exposure to As in groups with high milk consumption.

### Week Intake (WI), Dietary Risk Coefficient (DRC), Target Hazard Quotient (THQ), and Hazard Index (HI) to Pb, Cd, and As

The WI values for Pb and Cd in this study were similar to those reported by Amer et al. [79] in Alexandria, West Delta, Egypt, reported values of 1.05 and 0.11 μg/kg body weight, which contributed 4.2 and 1.6% of the TWI, respectively, which determined THQ < 1. In this study, the WI for As was higher than the TWI in those under 19 years. The DRCs for Pb and Cd by milk consumption, on all three consumption levels, were below 1, except for As.

Regarding THQ to Pb by milk consumption, our results are like those reported by Sharifi et al. [80] in different regions of the Tehran-Iran province, whose results showed THQ < 1 for all samples. Similar results are reported by Amer et al. [79] with THQs for Pb and Cd less than 1, indicative of safe consumption and not representing risk. A systematic review of studies of heavy metal health risks in China also indicates that the THQ for 11 toxic elements in milk, including Pb, Cd, and As, was less than 1, indicating negligible health risks.

In Guelma, Algeria, the THQ values for Pb and Cd suggest a potential infant’s risk. The HI was higher than 1, and the contributions of each metal to the HI generally followed a descending order for Pb, Cr, Cd, Ni, Zn, Cu, and Fe with values of 68.19%, 15.39%, 6.91%, 4.94%, 3.42%, 0.88%, and 0.28%, respectively, registering a potential risk of heavy metals, especially Pb, to infants through the consumption of raw cow milk [81].

Globally, very high levels of Pb in milk (60 mg/L) are reported in areas composed of granites and granite gneisses in India; the highest level of Cd (12 mg/L) was recorded in the barite mining area in India. In 10 regions out of 70, the THQs for Pb were >1, and in 6 out of 59, the THQ for Cd was > 1; this exposure is positively associated with the development of several fatal diseases [68].

Considering that the HI values at the three levels of intake were > 1 for those under 11 years of age, these results would determine that the milk produced in places close to mini mineral concentrators is not suitable for consumption, even more so if it is known that children consume more milk per kilogram of weight than adults. Therefore, the production of whole milk will be safe from an environmental point of view if systematic control of the farm environment is guaranteed, mainly by monitoring the content of As and heavy metals in irrigation water and soil. Dairy farms should not be contaminated with toxic elements, and studies such as this one provide profitable information to guide sustainable initiatives to control and evaluate environmental contamination. We recommended carrying out an update of the information and a permanent evaluation of the effects of heavy metals on human health.

### Study Limitations

Although the analysis of 19 milk samples could be considered a limitation of the study, these correspond to animals of the same herd, with the same food and health management, and with a similar physiological and productive state of the most representative farm in the area, thus controlling the animal effect. A strength of the study is that this farm adjoins a mini mineral concentrator and belongs to a joint property, so the information obtained offers evidence of food contamination in conditions of coexistence between livestock and mining activities in the Central Andes of Peru.

### Implications for Central Andes of Peru

Currently, the soils and grasslands of the Central Andes of Peru have high concentrations of heavy metals in other regions of the country and the world. There is a pattern of high heavy metal concentrations in the grasslands by the mining-metallurgical activity in the basin headwaters, whose emissions could lead to undesirable economic and social results. Our results and the reviewed scientific information suggest a possible trend between a higher intensity of mineral activity and the bioaccumulation of heavy metals. Additionally, the phosphorus fertilizer application also contributes to the accumulation of heavy metals in the soil, grass, and milk produced in the central highlands of Peru, entering the food chain and increasing the health risk due to its consumption.

The Pb at low levels and being a small fraction of the neurotoxic exposome would influence brain development, affecting neurological behavior. Joint exposure to Pb, Cd, and As can cause cognitive impairment and depressive disorders [82–84], and exposure to these toxins during pregnancy and lactation has neurological effects with lower neurobehavioral test results and reduced quality of life. These neurodevelopmental delays could be prevented or mitigated if public health policies are implemented to protect the fetus and young child from Pb, Cd, and As exposures.

Generalized anxiety disorders, panic disorder/agoraphobia, social anxiety disorder, and others are burdensome for communities [85–90]. This study expands the evidence to establish public health policies.

He et al. [91] found that increased blood Pb in children was associated with the impulsivity-hyperactivity index, anxiety, and attention deficit.

Studies carried out in Peru on mental health and its relationship with exposure to Pb, Cd, and As in children and adults surrounding the Las Bambas mining project in Apurímac report that before the exploitation phase, psychomotor development in <3 years of age, with the TEPSI test reporting a 12.5% risk in psychomotor development. The IQ with the Stanford-Binet test in children aged 3–12 years reports cases of mild mental retardation (2.1%) and borderline mental retardation [92].

This study highlights the association between the concentrations of heavy metals in the soil, pastures, and milk with the adjoining mineral processing mini-plants. There is a risk of bioaccumulation and possibly a potential risk to human health if more heavy metals and metalloids are evaluated since the emissions lead to the cocktail of contaminants introduced into the food chain. Therefore, it must generate a Peruvian guideline to control the Pb, Cd, and As in whole bovine milk levels.

## Conclusion

The consumption of milk produced in an area of the valley of the Central Andes adjacent to a small mineral concentrator, even when it has a lead and cadmium content above the permissible limits and an arsenic content close to 70% of the respective permissible limit, determined risk indices below 1, and there would be no health risk from its consumption in the Peruvian population between 2 and 85 years of age.

The milk Pb, Cd, and As content suggests that farms near small smelters do not produce environmentally safe and suitable milk for human consumption. Our findings can be used to develop guidelines to ensure milk production is safe and innocuous, fit for human consumption.

## References

[1] INDECOPI (2022). Informe Preliminar del Estudio de Mercado sobre el Sector Lácteo en el Perú.

[2] Midagri (2022) MIDAGRI promueve mayor consumo de leche para elevar calidad de la alimentación de población. https://bit.ly/3vQXRfE

[3] Minkina TM, Mandzhieva SS, Burachevskaya MV, Bauer TV, Sushkova SN (2018). Method of determining loosely bound compounds of heavy metals in the soil. Methods X.

[4] Su C, Gao Y, Qu X, Zhou X, Yang X, Huang S, Han L, Zheng N, Wang J (2021). The occurrence, pathways, and risk assessment of heavy metals in raw milk from industrial areas in China. Toxics.

[5] Zhou X, Zheng N, Su C, Wang J, Soyeurt H (2019). Relationships between Pb, As, Cr, and Cd in individual cows’ milk and milk composition and heavy metal contents in water, silage, and soil. Environ Pollut.

[6] Sharifi Z, Hossaini SMT, Renella G (2016). Risk assessment for sediment and stream water polluted by heavy metals released by a municipal solid waste composting plant. J Geochem Explor.

[7] Ibrahim MIA, Mohamed LA, Mahmoud MG, Shaban KS, Fahmy MA, Ebeid MH (2019). Potential ecological hazards assessment and prediction of sediment heavy metals pollution along the Gulf of Suez. Egypt Egypt J Aquat Res.

[8] Hameed A, Akhtar S, Amjad A, Naeem I, Tariq M (2019). Comparative assessment of arsenic contamination in raw milk, infant formulas and breast milk. Dairy and Vet Sci J.

[9] Castro-González NP, Calderón-Sánchez F, Pérez-Sato M, Soní-Guillermo E, Reyes-Cervantes E (2019). Health risk due to chronic heavy metal consumption via cow’s milk produced in Puebla, Mexico, in irrigated wastewater areas. Food Addit Contam Part B.

[10] Garaj-Vrhovac V, Zeljezic D (2002). Assessment of genome damage in a population of Croatian workers employed in pesticide production by chromosomal aberration analysis, micronucleus assay and Comet assay. J Appl Toxicol.

[11] Jafari A, Kamarehie B, Ghaderpoori M, Khoshnamvand N, Birjandi M (2018). The concentration data of heavy metals in Iranian grown and imported rice and human health hazard assessment. Data Brief.

[12] Tepanosyan G, Sahakyan L, Belyaeva O, Maghakyan N, Saghatelyan A (2017). Human health risk assessment and riskiest heavy metal origin identification in urban soils of Yerevan, Armenia. Chemosphere.

[13] González-Montaña JR, Senís E, Alonso AJ, Alonso ME, Alonso MP, Domínguez JC (2019). Some toxic metals (Al, As, Mo, Hg) from cow’s milk raised in a possibly contaminated area by different sources. Environ Sci Pollut Res Int.

[14] Ha TT, Tu VV, Tam KB, Ha TH (2019). Accumulation of arsenic and heavy metals in native and cultivated plant species in a lead recycling area in Vietnam. Minerals.

[15] Uddh-Söderberg TE, Gunnarsson SJ, Hogmalm KJ, Lindegård MMB, Augustsson ALM (2015). An assessment of health risks associated with arsenic exposure via consumption of homegrown vegetables near contaminated glassworks sites. Sci Total Environ.

[16] Chirinos-Peinado D, Castro-Bedriñana J, García-Olarte E, Quispe-Ramos R (2021). Gordillo-Espinal E (2021) Transfer of lead from soil to pasture grass and milk near a metallurgical complex in the Peruvian Andes. Transl Anim Sci.

[17] Codex Alimentarius Commission (2011). Report of the 50th Session of the Codex Committee on Food Additives and Contaminants.

[18] EFSA (2011). EFSA Panel on Contaminants in the Food Chain (CONTAM); Scientific opinion on tolerable weekly intake for cadmium, 2011. EFSA J.

[19] EFSA (2012). Lead dietary exposure in the European population. EFSA J.

[20] EFSA (2012). Cadmium dietary exposure in the European population (37 pp.). EFSA J.

[21] Anjos DC, Hernandez FF, Bañuelos GS, Dangi SR, Tirado-Corbalá R, da Silva FN, Filho PF (2018). Microbial community and heavy metals content in soils along the Curu River in Ceará, Brazil. Geoderma Reg.

[22] Latimer GW (2016). AOAC official method 973.35 lead in evaporated milk atomic absorption spectrophotometric method. Official methods of analysis of AOAC International.

[23] Alvira ML, Rada-Mendoza MDP, Hoyos SO, Villada CH (2012). Quantification of arsenic by atomic absorption spectrometry in flexible thermoformed and biodegradable films. Rev Bio Agro.

[24] Hashemi M (2018). Heavy metal concentrations in bovine tissues (muscle, liver, and kidney) and their relationship with heavy metal contents in consumed feed. Ecotoxicol Environ Saf.

[25] Martin AP, Turnbull RE, Rissmann CW, Rieger P (2017). Heavy metal and metalloid concentrations in soils under pasture of Southern New Zealand. Geoderma Reg.

[26] USEPA (1996). United States Environmental Protection Agency (USEPA) Acid digestion of sediments, sludges, and soils.

[27] Association of Official Analytical Chemists (AOAC) (1990). Association of Official Analytical Chemists (AOAC) AOAC-Official Method 975.03. Metals in plants: atomic absorption spectrophotometric method.

[28] Bidar G, Pruvot C, Garçon G, Verdin A, Shirali P, Douay F (2009). Seasonal and annual variations of metal uptake, bioaccumulation, and toxicity in *Trifolium repens* and *Lolium perenne* growing in a heavy metal-contaminated field. Environ Sci Pollut Res.

[29] Ramsumair A, Mlambo V, Lallo C (2014). Effect of drying method on the chemical composition of leaves from four tropical tree species. Trop Agric.

[30] Ministerio del Ambiente (MINAM) (2017) Decreto Supremo No. 011-2017-MINAM. Aprueban Estándares de Calidad Ambiental (ECA) para Suelo (Approval of Environmental Quality Standards (EQS) for Soil). https://sinia.minam.gob.pe/download/file/fid/64487. Accessed on 20 January 2022

[31] Canadian Council of Ministers of the Environment, CCME (2007) Canadian soil quality guidelines for the protection of environmental and human health. https://publications.gc.ca/collections/Collection/En1-34-9-2005E.pdf

[32] Fernández OB, Mullisaca CE, Huanchi ML (2022). Nivel de contaminación del suelo con arsénico y metales pesados en Tiquillaca (Perú). Revista de Investigaciones Altoandinas.

[33] Boularbah A, Schwartz C, Bitton G, Morel JL (2006). Heavy metal contamination from mining sites in South Morocco: 1. Use of a biotest to assess metal toxicity of tailings and soils. Chemosphere.

[34] Boularbah A, Schwart C, Bitton G, Aboudrar W, Ouhammou A, Morel JL (2006). Heavy metal contamination from mining sites in South Morocco: 2. Assessment of metal accumulation and toxicity in plants. Chemosphere..

[35] Kabata-Pendias A, Mukherjee AB (2007). Trace elements from soil to human.

[36] OJEU (2013) Commission Regulation (EU) No 1275/2013. Off J Eur Union. https://acortar.link/OssuRK

[37] CENAN-INEI (2011). Estado Nutricional en el Perú. Componente Nutricional ENAHO-CENANz-INS.

[38] Christophoridis C, Kosma A, Evgenakis M, Bourliva A (2019). Determination of heavy metals and health risk assessment of cheese products consumed in Greece. J Food Compos Anal.

[39] Khan K, Khan H, Lu Y, Ihsanullah I, Nawab J, Khan S, Shah NS, Shamshad I, Maryam A (2014). Evaluation of toxicological risk of foodstuffs contaminated with heavy metals in Swat, Pakistan. Ecotoxicol Environ Saf.

[40] JECFA. Joint FAO/WHO Expert Committee on Food Additives (2011). Evaluation of certain food additives and contaminants. 73 Report, 2010; Technical Report Series.

[41] Joint and World Health O (2012). Safety evaluation of certain food additives and contaminants: prepared by the seventy fourth meeting of the Joint FAO/WHO Expert Committee on Food Additives (JECFA).

[42] USEPA (2020). Regional screening levels (RSLs) - generic tables. Regional screening level (RSL) summary table (TR=1E-06, HQ=0.1).

[43] Agostoni C, Turck D (2011). Is cows’ milk harmful for a childʼs health?. J Pediatr Gastroenterol Nutr.

[44] Daniels SR, Hassink SG (2015). Committee on nutrition. The role of the pediatrician in primary prevention of obesity. Pediatrics.

[45] Dror DK, Allen LH (2013). Dairy product intake in children and adolescents in developed countries: trends, nutritional contribution, and a review of association with health outcomes. Nutr Rev.

[46] Grenov B, Larnkjær A, Mølgaard C, Michaelsen KF (2020). Role of milk and dairy products in growth of the child. Nestle Nutr Inst Workshop Ser.

[47] Singh GM, Micha R, Khatibzadeh S, Shi P, Lim S, Andrews KG, Engell RE, Ezzati M, Mozaffarian D (2015). Global, regional, and national consumption of sugar-sweetened beverages, fruit juices, and milk: a systematic assessment of beverage intake in 187 countries. PloS One.

[48] USDA (2020) Dairy update. Country: Peru. United States Department of Agricultura. Foreing Agricultural Service. Report number: PE2020–0024. https://www.fas.usda.gov/data/peru-dairy-update.

[49] Jin Y, Liu P, Sun J, Wang C, Min J, Zhang Y, Wang S, Wu Y (2014). Dietary exposure and risk assessment to lead of the population of Jiangsu province, China. Food Addit Contam Part A.

[50] Juric AK, Batal M, David W, Sharp D, Schwartz H, Ing A, Fediuk K, Black A, Tikhonov C, Chan HM (2018). Risk assessment of dietary lead exposure among First Nations people living on-reserve in Ontario, Canada using a total diet study and a probabilistic approach. J Hazard Mater.

[51] Rahmani J, Fakhri Y, Shahsavani A, Bahmani Z, Urbina MA, Chirumbolo S (2018). A systematic review and meta-analysis of metal concentrations in canned tuna fish in Iran and human health risk assessment. Food Chem Toxicol.

[52] Castro Gonzalez NP, Moreno-Rojas R, Calderón SF (2017). Assessment risk to children’s health due to consumption of cow’s milk in polluted areas in Puebla and Tlaxcala, Mexico. Food Addit Contam Part B Surveill.

[53] USEPA (2011) USEPA regional screening level (RSL) summary table: November 2011

[54] USEPA (2012) EPA region III risk-based concentration (RBC) table 2008 region III, 1650 Arch Street, Philadelphia, Pennsylvania 19103

[55] Liu X, Song Q, Tang Y, Li W, Xu J, Wu J, Wang F, Brookes PC (2013). Human health risk assessment of heavy metals in soil-vegetable system: a multi-medium analysis. Sci Total Environ.

[56] Norouzirad R, González-Montaña JR, Martínez-Pastor F, Hosseini H, Shahrouzian A, Khabazkhoob M, Malayeri FA, Bandani H, Paknejad M, Foroughi-Nia B (2018). Lead and cadmium levels in raw bovine milk and dietary risk assessment in areas near petroleum extraction industries. Sci Total Environ.

[57] Castro J, Chirinos-Peinado D, Peñaloza R (2020). Lead bioaccumulation in root and aerial part of natural and cultivated pastures in highly contaminated soils in Central Andes of Peru. Advances in Science Technology and Engineering Systems Journal.

[58] Hou Q, Yang Z, Ji J, Yu T, Chen G, Li J, Xia X, Zhang M, Yuan X (2014). Annual net input fluxes of heavy metals of the agro-ecosystem in the Yangtze River delta, China. J Geochem Explor.

[59] Khan S, Cao Q, Zheng YM, Huang YZ, Zhu YG (2008). Health risks of heavy metals in contaminated soils and food crops irrigated with wastewater in Beijing, China. Environ Pollut.

[60] Kozhanova N, Sarsembayeva N, Lozowicka B, Kozhanov Z (2021). Seasonal content of heavy metals in the “soil-feed-milk-manure” system in horse husbandry in Kazakhstan. Vet World.

[61] Soto-Benavente M, Rodriguez-Achata L, Olivera M, Arostegui SV, Colina NC, Garate QJ (2020). Health risks due to the presence of heavy metals in agricultural products cultivated in areas abandoned by gold mining in the Peruvian Amazon. Scientia Agropecuaria.

[62] Schipper LA, Sparling GP, Fisk LM, Dodd MB, Power IL, Littler RA (2011). Rates of accumulation of cadmium and uranium in a New Zealand hill farm soil as a result of long-term use of phosphate fertilizer. Agric Ecosyst Environ.

[63] Chirinos-Peinado D, Castro-Bedriñana J, Ríos-Ríos E, Mamani-Gamarra G, Quijada-Caro E, Huacho-Jurado A, Nuñez-Rojas W (2022). Lead and cadmium bioaccumulation in fresh cow’s milk in an intermediate area of the Central Andes of Peru and risk to human health. Toxics..

[64] Zhang F, Li Y, Yang M, Li E (2012). Content of heavy metals in animal feeds and manures from farms of different scales in Northeast China. Int J Environ Res Public Health.

[65] ATSDR (2020). Substance priority list; Agency for Toxic Substances and Disease Registry: Atlanta, GA, USA, 2020.

[66] European-Union (2015). Commission Regulation (EU) 2015/1005 of 25 June 2015 amending Regulation (EC) N° 1881/2006 as regards maximum levels of lead in certain foodstuffs. Off J Eur Union.

[67] Food and Agriculture Organization of the United Nations (2013). Codex Alimentarius Commission. The Secretariat of the Joint FAO/WHO Food Standards Program.

[68] Boudebbouz A, Boudalia S, Bousbia A, Habila S, Boussadia MI, Gueroui Y (2021). Heavy metals levels in raw cow milk and health risk assessment across the globe: a systematic review. Sci Total Environ.

[69] Shahbazi Y, Ahmadi F, Fakhari F (2016). Voltammetric determination of Pb, Cd, Zn, Cu, and Se in milk and dairy products collected from Iran: an emphasis on permissible limits and risk assessment of exposure to heavy metals. Food Chem.

[70] Diacono E, Faye B, Meldebekova A, Konuspayeva G, Faye B, Sinyavskiy Y (2008). Plant, water and milk pollution in Kazakhstan. Impact of pollution on animal products. NATO Science for Peace and Security Series Series C: Environmental Security.

[71] Eleboudi AA, El-Makarem HA, Hadour HH (2017). Heavy metals residues in some dairy products. Alexandria J Vet Sci.

[72] Ayala J, Romero H (2013). Presencia de metales pesados (arsénico y mercurio) en leche de vaca al sur de Ecuador. Revista de Ciencias de la vida.

[73] Pérez-Carrera A, Fernández-Cirelli A (2005). Arsenic concentration in water and bovine milk in Cordoba, Argentina. Preliminary results. J Dairy Res.

[74] Licata P, Trombetta D, Cristani M, Giofrè F, Martino D, Calò M, Naccari F (2004). Levels of “toxic” and “essential” metals in samples of bovine milk from various dairy farms in Calabria, Italy. Environ Int.

[75] Arianejad M, Alizadeh M, Bahrami A, Arefhoseini SR (2015). Levels of some heavy metals in raw cow’s milk from selected milk production sites in Iran: is there any health concern?. Health Promot Perspect.

[76] Su C, Liu H, Qu X, Zhou X, Gao Y, Yang H, Zheng N, Wang J (2020). Heavy metals in raw milk and dietary exposure assessment in the vicinity of leather-processing plants. Biol Trace Elem Res.

[77] Ali H, Khan E, Sajad MA (2013). Phytoremediation of heavy metals-concepts and applications. Chemosphere.

[78] Rosas JM, Guzmán JL, Hernández A, Garza MT, Hinojosa L (2014). Arsenic accumulation in maize crop (Zea mays): a review. Sci Total Environ.

[79] Amer AAE, El-Makarem HSA, El-Maghraby MA, Abou-Alella SA (2021). Lead, cadmium, and aluminum in raw bovine milk: residue level, estimated intake, and fate during artisanal dairy manufacture. J Adv Vet Anim Res.

[80] Sharifi S, Sohrabvandi S, Mofid V, Javanmardi F, Khanniri E, Mortazavian AM (2022). The assessment of lead concentration in raw milk collected from some major dairy farms in Iran and evaluation of associated health risk. J Environ Health Sci Eng.

[81] Boudebbouz A, Boudalia S, Bousbia A, Gueroui Y, Boussadia MI, Chelaghmia ML, Zebsa R, Affoune AM, Symeon GK (2022). Determination of heavy metal levels and health risk assessment of raw cow milk in Guelma Region, Algeria. Biol Trace Elem Res.

[82] Ramírez OD, González EDF, Blanco AT, Pineda B, Gómez MS, Quino MJ, Carrillo MP, Pérez de la Cruz V (2021). Cognitive impairment induced by lead exposure during lifespan: mechanisms of lead neurotoxicity. Toxics.

[83] Skogheim TS, Vegard FK, Weyde EMS, Aase H, Surén P, Øie GM, Biele G, Reichborn-Kjennerud T, Caspersen HI, Hornig M, Haug SL, Villanger DG (2021). Metal and essential element concentrations during pregnancy and associations with autism spectrum disorder and attention-deficit/hyperactivity disorder in children. Environ Int.

[84] Zaw YH, Taneepanichskul N (2019). Blood heavy metals and brain-derived neurotrophic factor in the first trimester of pregnancy among migrant workers. PloS One.

[85] Ayuso-Álvarez A, Simón L, Nuñez O, Rodríguez-Blázquez C, Martín-Méndez I, Bel-Lán A (2019). Association between heavy metals and metalloids in topsoil and mental health in the adult population of Spain. Environ Res.

[86] Berk M, Williams LJ, Andreazza AC, Pasco JA, Dodd S, Jacka FN (2014). Heavy metal and the blues: secondary analysis of persistent organic pollutants (POP), heavy metals and depressive symptoms in the NHANES National Epidemiological Survey. BMJ Open.

[87] Jaga K, Dharmani C (2007). The interrelation between organophosphate toxicity and the epidemiology of depression and suicide. Rev Environ Health.

[88] Jurczak A, Brodowska A, Szkup M, Prokopowicz A, Karakiewicz B, Łój B (2018). Influence of Pb and Cd levels in whole blood of postmenopausal women on the incidence of anxiety and depressive symptoms. Ann Agric Environ Med.

[89] Theorell T, Hammarström A, Aronsson G, Träskman Bendz L, Grape T, Hogstedt C (2015). A systematic review including meta-analysis of work environment and depressive symptoms. BMC Public Health.

[90] WHO (2017). Depression and other common mental disorders: global health estimates.

[91] He B, Wang Y, Li S, Zhao Y, Ma X, Wang W, Li X, Zhang Y (2021). A cross–sectional survey of preschool children: exploring heavy metal exposure, neurotransmitters, and neurobehavioural relationships and mediation effects. Ecotoxicol Environ Saf.

[92] Astete J, Gastañaga MC, Fiestas V, Oblitas T, Sabastizagal I, Lucero M, Abadíe JM, Muñoz ME, Valverde A, Suarez M (2010). Comunicable diseases, mental health and exposure to environmental pollutants in population living near Las Bambas mining project before exploitation phase, Peru 2006. Rev Peru Med Exp Salud Publica.

